# Utilization of Patient-Generated Data Collected Through Mobile Devices: Insights From a Survey on Attitudes Toward Mobile Self-Monitoring and Self-Management Apps for Depression

**DOI:** 10.2196/11671

**Published:** 2019-04-03

**Authors:** Ralf Hartmann, Christian Sander, Noah Lorenz, Daniel Böttger, Ulrich Hegerl

**Affiliations:** 1 Research Center of the German Depression Foundation Leipzig Germany; 2 Department of Psychiatry University of Leipzig Leipzig Germany

**Keywords:** mHealth, depression, adherence, mobile applications, self-management

## Abstract

**Background:**

Depression is a severe psychiatric disease with high prevalence and an elevated risk for recurrence and chronicity. A substantial proportion of individuals with a diagnosis of unipolar depressive disorder do not receive treatment as advised by national guidelines. Consequently, self-monitoring and self-management become increasingly important. New mobile technologies create unique opportunities to obtain and utilize patient-generated data. As common adherence rates to mobile technologies are scarce, a profound knowledge of user behavior and attitudes and preferences is important throughout any developmental process of mobile technologies and apps.

**Objective:**

The aim of this survey was to provide descriptive data upon usage and anticipated usage of self-monitoring and self-management of depression and preferences of potential users in terms of documented parameters and data-sharing options.

**Methods:**

A Web-based survey comprising 55 questions was conducted to obtain data on the usage of mobile devices, app usage, and participant’s attitudes and preferences toward mobile health apps for the self-monitoring and self-management of depression.

**Results:**

A total of 825 participants provided information. Moreover, two-thirds of the sample self-reported to be affected by depressive symptoms, but only 12.1% (81/668) of those affected by depression have ever used any mobile self-monitoring or self-management app. Analysis showed that people want personally relevant information and feedback but also focus on handling sensitive data.

**Conclusions:**

New mobile technologies and smartphone apps, especially in combination with mobile sensor systems, offer unique opportunities to overcome challenges in the treatment of depression by utilizing the potential of patient-generated data. Focus on patient-relevant information, security and safe handling of sensitive personal data, as well as options to share data with self-selected third parties should be considered mandatory throughout any development process.

## Introduction

Depression is a severe disease with large effects on well-being and quality of life [1]. Major depressive disorder (MDD) is highly prevalent [2-5] and is a prime cause for years lived with disability [6] and a major source of the global burden of disease [1,7]. Furthermore, MDD is associated with a high risk of recurrence and chronicity [8]. Although diagnostics and evidence-based treatments (eg, pharmacotherapy, psychotherapy) for depressive disorders [9-12] are available, a substantial proportion of individuals with a diagnosis of unipolar depressive disorder do not receive treatment as advised by national guidelines [13]. Consequently, self-monitoring and self-management become ever more important. The opportunities that have arisen from the digital and mobile revolution of recent years [14] bear the potential to meet these challenges. Mobile devices such as smartphones or wearable biosensors can assess and record multimodal data such as physiological data, self-ratings, user behavior, or environmental data. Such patient-generated data become increasingly available and have promising potential to be utilized for self-monitoring, self-management, and medical care. However, this field of research is young and it displays much dynamic [15]. Throughout any process of development or implementation of mobile systems or apps for self-monitoring and self-management, a profound knowledge of readiness for use and user behavior is necessary [16] as common adherence to mobile health (mHealth) systems or apps is often weak [17]. So far, only a few studies have explored preferences and usage of mHealth apps in general [18,19] or for specific fields of interest [20] but not for depression. The aim of this survey was to provide descriptive data to answer the following questions: to what extent are mobile apps for the self-monitoring and self-management of depression (for the purpose of readability, the following abbreviation is used henceforth: MSSD) currently used, what is the anticipated future use, and what do potential users prefer in terms of documented parameters and data-sharing options.

## Methods

### Survey

We conducted a Web-based comprising 55 questions survey using Unipark, an Web-based survey tool to program and evaluate surveys for academic research provided by Questback GmbH. The survey was available from January 2017 to March 2017. It was accessible via any internet browser on stationary and mobile devices. It was hosted on the servers of Unipark. The internal procedures of Unipark for tests of consistency were used. The link to the survey was prominently posted on the website of the German Depression Foundation (GDF), the “Depressionsforum,” a discussion forum about depression-related topics, which is run by the GDF. GDF is a nonprofit organization in Germany to promote information, knowledge and acceptance of depression, and treatment options within Germany. It was also attached to newsletters of the GDF during the period the survey was available.

### Sample

A total of 1174 participants commenced the survey. Of them, 159 participants were excluded because they did not complete the survey, and in addition, 17 participants were excluded because of missing age data. The remaining sample consisted of 998 participants with a mean age of 38.29 (SD 12.358, range 18-84) years. Gender was not distributed evenly, with 67.2% (671/998) of the sample being female. Of them, 668 participants indicated by self-rating to have been diagnosed with unipolar depressive disorder at least once in their life. This subsample of affected individuals was included in subsequent analyses.

### Procedure

The survey was adaptive in a way that depending on individual responses, participants were provided specific sections of the questionnaire. The main filter question was a self-rating question enquiring about whether or not individuals suffer from depressive symptoms at the moment or if they did ever before (for German questionnaire, see Multimedia Appendix 1). To address our research questions, we provided different blocks of questions. The first block of question assessed the mere usage and duration of usage of MSSDs by self-rating. Individuals who responded with no to the first block were provided separate questions to assess their self-rated anticipated future usage of MSSDs. The second block of questions assessed the mere usage and duration of usage of wearables (eg, fitness tracker or any product of similar functionality to monitor physiological parameters such as skin conductance, heart rate, temperature, or position) by self-rating. Individuals who responded with no were provided separate questions to assess their self-rated anticipated future usage of wearables. The penultimate block of questions assessed what parameters participants would prefer to be documented by MSSDs regardless of the method of data collection (eg, self-rating, sensor-based automatic recording). We provided categories of distinct parameters from which individuals could pick their respective favorites by multiple choice. The last block of questions assessed with whom participants might want to share data for specific purposes. We provided distinct groups of social agents from which individuals could pick their respective favorites by multiple choice.

### Statistical Analyses

SPSS (SPSS 24, IBM, Chicago, IL, USA) was used to perform statistical analyses. Descriptive analyses and Chi-square tests were performed to address our research questions.

## Results

### Demographics of the Sample of Affected Individuals

Demographics of the analyzed sample of affected individuals can be found in Table 1. Within the affected sample, the proportion of women was at 76.9% (514/668). There were significant gender differences for the self-estimated duration of smartphone usage per day (Χ^2^_3_=13.1; *P*=.005). More women estimated their smartphone usage duration per day to be more than 2 hours compared with men. We found no age differences and collapsed age for subsequent analyses. We found no significant group differences for demographics between nondepressive individuals and depressive individuals. We found no significant group differences for demographics between users of MSSDs or wearables and individuals who did not use MSSDs or wearables.

**Table 1:** Characteristics and descriptive data of the subsample of affected individuals (N=668).

Characteristics		Statistics
**Gender, n (%)**		
	Female	514 (76.9)
	Male	154 (23.1)
Age (years), mean (SD)		39.31 (12.857)
**Occupation, n (%)**		
	Employed	334 (50.0)
	Self-employed	26 (3.9)
	In education: completing education	87 (13.0)
	Taking care of household	27 (4.0)
	Retired	88 (13.2)
	Unemployed	70 (10.5)
	Other	36 (5.4)
**Relationship, n (%)**		
	Single	262 (39.2)
	Married	347 (52.0)
	Other	59 (8.8)

**Table 2:** Self-reported duration of usage of mobile apps for the self-monitoring and self-management of depression in the subsample of affected individuals (n=111; 1=less than 1 month, 2=more than 1 month).

“For how long do you use the app?”	Relative amount of entries, n (%^a^)
Installed but discarded	24 (21.6)
Installed for a short (1) period, used irregularly	29 (26.1)
Installed for a short (1) period, used regularly	16 (14.4)
Installed for a long (2) period, used irregularly	8 (7.2)
Installed for a long (2) period, used regularly	28 (25.2)
Provided categories not applicable	6 (5.4)

^a^Percentages do not sum up to 100% due to rounding errors.

### Usage and Anticipated Future Usage of Mobile Apps for the Self-Monitoring and Self-Management of Depression

Within the affected sample, a proportion of 81 individuals (12.1%, 81/668) reported to have installed and used an MSSD at least once in their life. Moreover, 24 individuals installed an MSSD but did not use it (3.6%, 24/668). The majority of the subsample of affected individuals (78.0%, 521/668) stated to have never used any respective app. For the duration of usage, see Table 2. From those who did not use any app at the moment, a total of 391 (75.1%, 391/521) individuals could imagine using any such app in the future (Table 3).

### Usage and Anticipated Future Usage of Wearables

From the affected sample, a proportion of 94 individuals (14.1%, 94/668) indicated to use wearables, 553 (82.8%, 553/668) reported no prior usage, and 21 individuals refused to answer. For period of usage of wearables throughout a day, see Table 4; for the duration of usage of wearables, see Table 5. From the subsample of nonusers of wearables, 71.2% (394/553) could imagine doing so in the future at least sometimes. However, when people were asked at what time during the day they would usually wear such wearables, responses are heterogeneous (see Table 6).

Only a small proportion of individuals from the whole sample indicated to have used a wearable and an MSSD simultaneously (3.6%, 24/668). The majority of individuals who indicated to have used a wearable before had never used an MSSD before (74%, 70/94).

### Preferences for Documentable Parameters

Categories of documentable parameters and results are presented in Table 7. Low preferences were found for 2 categories: 502 (75.1%, 502/668) individuals reported to not prefer any tracking of location and movement by Global Positioning System (GPS) and 439 (65.7%, 439/668) individuals reported to not prefer any tracking of social interaction or communication.

**Table 3:** Self-reported anticipated future use by nonusers of mobile apps for the self-monitoring and self-management of depression (MSSDs) in the subsample of affected individuals (n=521).

“Can you imagine using a MSSD in the future?”	Relative amount of entries, n (%)
I can imagine using such an app	391 (75.1)
I do not know	120 (23.0)
No, I would not use such an app	10 (1.9)

**Table 4:** Self-reported period of usage of wearables throughout 1 day for those that own wearables in the subsample of affected individuals (n=94).

“When do you use a fitness tracker?”	Relative amount of entries, n (%^a^)
Only for sports	11 (11.7)
Only at daytimes	29 (30.9)
Only at night	1 (1.1)
All the time	53 (56.4)

^a^Percentages do not sum up to 100% due to rounding errors.

**Table 5:** Self-reported duration of usage of wearables for those that own wearables in the subsample of affected individuals (n=94).

“For how long did you use a fitness tracker?”	Relative amount of entries, n (%)
A few days	12 (12.8)
A few weeks	21 (22.3)
A few months	61 (64.9)

**Table 6:** Self-reported anticipated future use by nonusers of wearables in the subsample of affected individuals (n=553).

“When would you use a fitness tracker?”	Relative amount of entries, n (%^a^)
Only for sports	71 (12.8)
Only at daytime	153 (27.7)
Only at night	7 (1.3)
All the time	163 (29.5)
Never	159 (28.8)

^a^Percentages do not sum up to 100% due to rounding errors.

**Table 7:** Self-reported preferred category options of documentable parameters in the subsample of affected individuals (N=668).

“Which options of documentable parameters would you use?”	Agreement, n (%)	Disagreement, n (%)
Mood	568 (85.0)	100 (15.0)
Stress	507 (75.9)	161 (24.1)
Sleep	479 (71.7)	189 (28.3)
Goals	451 (67.5)	217 (32.5)
Sports	427 (63.9)	241 (36.1)
Medication	331 (49.6)	337 (50.4)
Social interaction	229 (34.3)	439 (65.7)
Location (GPS^a^)	166 (24.9)	502 (75.1)

^a^GPS: Global Positioning System.

**Table 8:** Self-reported preferences for data-sharing options in the subsample of affected individuals (N=668).

“With whom would you share personal data?”	Agreement, n (%)	Disagreement, n (%)
Science	417 (62.4)	251 (37.6)
Psychotherapist	417 (62.4)	251 (37.6)
Psychiatrist	345 (51.6)	323 (48.4)
General practitioner	294 (44.0)	374 (56.0)
Family	126 (18.9)	542 (81.1)
Other users of the app	124 (18.6)	544 (81.4)
Friends	85 (12.7)	583 (87.3)
Health insurance companies	30 (4.5)	638 (95.5)

### Preference for Data-Sharing Options

The frequencies of the different categories of data sharing options are presented in Table 8. Low preferences were found for sharing data with health insurance companies (4.5%, 30/668) and sharing data with friends (12.7%, 85/668). We found significant gender differences for category “General Practitioner” (Χ^2^_1_=9.0; *P*=.003) and for category “psychiatrist” (Χ^2^_1_=12.8; *P*<.001).

## Discussion

### Principal Findings

For a successful utilization of the multitude of patient-generated data through mobile devices, patients often need to generate monotonous and repetitive data over a long period. To ameliorate their motivation and engagement to do so, it is inevitable to respond to their readiness, user behavior, and preferences.

### Usage and Anticipated Future Usage of Mobile Apps for the Self-Monitoring and Self-Management of Depression

Usage of MSSDs was scarce within our sample. Although 30% of individuals used an MSSD for at least 1 month or longer on a regular basis, the majority of the sample used it for shorter periods and irregularly. Despite perceived and anticipated benefits of smartphone apps for the self-monitoring and self-management of depression and/or wearable fitness trackers to support and shape attempts of self-monitoring and self-management, the mere usage of such instruments is still poor. Even if such digital helpers have been used once, duration of usage is mostly limited, and only a small proportion uses them constantly and regularly. This might have different reasons. First of all, the market of mHealth apps is diverse, and finding suitable options can be confusing. Yet, information about evidence-based effects is scarce. Interested users might find it difficult to decide upon which program might fit their own specific needs. An inappropriate choice might disengage further motivation to search for and use MSSDs. Recently proposed guidelines for the evaluation and informed decision-making might help overcome those limitations [21]. Second, mHealth apps often require a constant stream of data and information input to work properly and provide support. Repetitive and monotonous data input for a long period might result in motivational losses as individuals’ prior drive declines after they started a program. Developers might thrive for an appetizing user interface and data acquisition mechanisms to maintain individuals’ drive until it is integrated into their daily routine.

### Usage and Anticipated Future Usage of Wearables

Within our sample, usage of wearables was scarce. Only a small proportion of those who use wearables applied them to their daily routine throughout 24 hours a day. This might be because of different reasons. Wearables have advantages as well as disadvantages with respect to different aspects such as reliability or energy management [22]. Potential users also have individual preferences in terms of design, color, haptics, and weight. Most individuals did not use wearables and MSSDs together. Wearables might be perceived as gadgets for personal activity or sports but not as part of systems to support self-monitoring and self-management of depression. However, individual responses to anticipated future use are heterogeneous.

### Preferences for Documentable Parameters

This survey assessed information about individual preferences and expectations people would want from digital assistants for self-monitoring and self-management of depression. The possibilities to document daily mood, personal goals, sports, sleep, and stress level receive broad agreement. The documentation of medication was only important to half the sample. This is maybe more a question of practicality than a question of desire. Less than a third of our sample agreed to the documentation of social interaction. Individuals do not favorably evaluate gathering data about their communication behavior with others, which includes communication channels, number of communication partners, time and amount of communication, as well as content and quality of social interaction. One possible explanation might be that individuals do not expect meaningful outcomes from such data. Moreover, these findings might partly reflect the individual strive to booster their self-esteem and their denial of contrasting or interfering information to that [23,24]. They might not want to be confronted with difficulties and failures of their social interactions and communication behavior. They also might want to avoid actualizing unpleasant interactions and experiences. Detailed information about one’s own communication behavior and alterations and suggested modification based on such information interfere with one’s own perception of the self as a competent agent. This might be of particular interest for the research of self-enhancement and self-improvement states and traits [25]. More interestingly, we found that individuals have specific issues when it comes to tracking of location data via GPS, accelerometers, and gyroscopes. Within our sample, the majority of the people do not want to be tracked in detail where they are or where they were. However, this finding has to be considered carefully. It does not mean that individuals do not want tracking in general. However, individuals want to keep control of such sensitive data and just do not want to share it with everybody or more precisely with third-party agents from whom negative consequences could arise from, such as German public health insurance, for instance. People are worried about being tracked at places that indicate risk behavior or self-damaging behavior, which could result in financial consequences (eg, higher insurance rates or loss of treatment reimbursement). Furthermore, people do not want to share tracking data with commercial agents because they do not want to be spammed with advertising, unwanted offers, or customized products. People are interested in tracking and analyzing tracked location data for their own and privileged purpose to find correlations with their idiosyncratic disease. We interpret the findings as an individual desire for privacy and control of personal data. Hence, sensitive data should be stored securely in devices the individual has control of, and they can voluntarily decide with whom to share this information.

### Preference for Data-Sharing Options

There is agreement on sharing data with professionals such as physicians, psychotherapists, and scientists. As gathering data requires detailed, precise, and reliable analysis and interpretation, there is a strong need for expertise and well-trained personnel to provide such service. In the field of psychiatric disorders and psychological conditions, this expertise is usually delivered by mental health professionals, respectively, psychiatrists and/or psychotherapists. There seems to be no difference if the professional is a psychiatrist, a psychotherapist, or a general practitioner. Individuals know that professionals need information to decide upon medication and psychotherapeutic treatments and therefore accept the disclosure of sensitive data. Moreover, men quoted medical professionals (general practitioner, psychiatrist) more often than women as a sharing option. However, against the odds, people also do not want their family and close friends to know about where they are. This might be partly reasoned by the fact that social interactions even with close friends are characterized by at least a minimum of specific undisclosed aspects of their life [26,27]. Individuals might be afraid to reveal disclosures, such as places, habits, or activities, to avoid effects on self-esteem or maintain control [28,29].

### Conclusions and Limitations

Nonetheless, our survey embodies some important limitations. First, we do not have a representative sample because of the biased distribution via the networks of the GDF solely through internet-based pathways. Furthermore, the limitations that come with Web-based surveys might corrupt our results. To be specific, this means that we took much effort to prevent double entries, fake entries, or robot entries. However, there is no chance of keeping results totally clear of such influences. Another aspect that confounds our results is that we used precategorized questions to address our questions. This simplifies answers and reduces variance. We used an adaptive structure for the survey to comfort the user. This might limit comparisons between groups. Gender distribution within the sample was skewed, and more women answered the questionnaire. From the literature, we expected to find women to be overrepresented in the affected individuals’ sample [2,4]. The gender balance within the respective subsample of affected individuals was at an expected ratio of 1:3. Finally, we did not assess information about future usage and continuation of usage and what reasons lead to discontinuation.

This survey provides information about usage and preferences toward eHealth app for the self-monitoring and self-management of depression. New mobile technologies and smartphone apps, especially in combination with mobile sensor systems, offer unique opportunities to overcome challenges in the treatment of depression by utilizing the potential of patient-generated data. Throughout any development process of such mobile smartphone apps or systems, a focus on patient-relevant data, security and safe handling of sensitive personal data, as well as degrees of freedom to share data with self-selected third parties should be considered mandatory. This can melt down barriers, make digital helpers much more attractive, and consequently sustain and ameliorate adherence.

## Appendix

Multimedia Appendix 1 Patientenbefragung, Web-based German Questionnaire.

## Abbreviations

GDF: German Depression Foundation
GPS: Global Positioning System
MDD: major depressive disorder
mHealth: mobile health
MSSD: mobile apps for the self-monitoring and self-management of depression
