# Supplementary material for: Utilization of Patient-Generated Data Collected Through Mobile Devices: Insights From a Survey on Attitudes Toward Mobile Self-Monitoring and Self-Management Apps for Depression
Source: JMIR Ment Health. 2019 Apr 3;6(4):e11671. doi: 10.2196/11671 (PMC6468343; doi:10.2196/11671)
Supplement: Multimedia Appendix 1 [file mental_v6i4e11671_app1.pdf]

Befragung „Depressions-Apps“

Smartphones und Apps sind aus dem Alltag heute nicht mehr wegzudenken. Zunehmend werden auch Apps entwickelt, die helfen sollen, Depressionen zu bewältigen.
Die Stiftung Deutsche Depressionshilfe möchte solche „Depressions-Apps“ erforschen und entwickeln. Dies erfolgt im Rahmen des Forschungsprojektes “STEADY”, das durch das Bundesministerium für Bildung und Forschung (BMBF) gefördert wird (Förderkennzeichen: 13GW0162B). Dabei sollen von Beginn an Erwartungen und Erfahrungen potenzieller Nutzer berücksichtigt werden.
Bitte helfen Sie uns mit ein paar Minuten Ihrer Zeit, um die folgenden Fragen zu beantworten. Dabei gibt es kein richtig oder falsch: Wählen Sie einfach jeweils die passendste Antwort aus. Wir sind für Ihre Mithilfe sehr dankbar.
Diese Befragung richtet sich ausschließlich an volljährige Personen. Wenn Sie das 18. Lebensjahr noch nicht vollendet haben, nehmen Sie bitte nicht teil.
Die Umfrage findet natürlich VOLLSTÄNDIG ANONYM statt. Es werden keine persönlichen Daten gespeichert, die Rückschlüsse auf Sie als Person zulassen.
Vielen Dank!
Prof. Ulrich Hegerl & das STEADY-Team

1. Sind Sie mit dem Thema Depression bereits in Kontakt gekommen?
Wenn mehr als eine Antwortoption auf Sie zutrifft, wählen Sie bitte die Option, die Sie für sich am passendsten erachten!

- ☐ Ja, ich bin/war selbst erkrankt
- ☐ Ja, ein Angehöriger/Bekannter ist/war erkrankt
- ☐ Ja, ich behandle/berate Menschen mit Depressionen
- ☐ Ich habe keine direkte Verbindung zum Thema Depression
- ☐ Sonstiger Kontakt

2. Wie oft fühlten Sie sich im Verlauf der letzten 2 Wochen durch die folgenden Beschwerden beeinträchtigt?

Wenig Interesse oder Freude an Ihren Tätigkeiten

- ☐ Überhaupt nicht
- ☐ An einzelnen Tagen
- ☐ An mehr als der Hälfte der Tage
- ☐ Beinahe jeden Tag

Niedergeschlagenheit, Schwermut oder Hoffnungslosigkeit

- ☐ Überhaupt nicht
- ☐ An einzelnen Tagen
- ☐ An mehr als der Hälfte der Tage
- ☐ Beinahe jeden Tag

3. Besitzen und benutzen Sie ein einziges Smartphone oder mehrere?

- ☐ Ich besitze und benutze nur ein Smartphone
- ☐ Ich besitze und benutze mehrere Smartphones parallel
- ☐ Ich besitze kein Smartphone

4. Von welchem Hersteller ist Ihr Smartphone?

- ☐ Apple
- ☐ Honor
- ☐ HTC
- ☐ Huawei
- ☐ LG
- ☐ Microsoft (Windows Phone)
- ☐ Nokia
- ☐ Samsung
- ☐ Sony
- ☐ Umi
- ☐ Sonstiger Hersteller
- ☐ weiß ich nicht

Wie häufig wird Ihr Smartphone von anderen Personen verwendet?

- ☐ Nie
- ☐ Sehr selten
- ☐ Gelegentlich
- ☐ Häufig

Haben Sie eine permanente Internetverbindung mit Ihrem Smartphone?

- ☐ Ja, mit einer Handyflatrate
- ☐ Ja, aber ohne Flatrate (ich zahle pro Nutzung)
- ☐ Nein, ich gehe nur über W-Lan mit dem Smartphone ins Internet
- ☐ Mein Smartphone hat keine Verbindung zum Internet
- ☐ Anderes: ...

Bitte schätzen Sie: Wie oft am Tag schauen Sie ungefähr auf Ihr Smartphone?

- ☐ Weniger als 5 mal.
- ☐ 5 bis 20 mal.
- ☐ 21 bis 50 mal.
- ☐ 51 bis 100 mal.
- ☐ Mehr als 100 mal.

Bitte schätzen Sie: Wieviel Zeit am Tag verbringen Sie mit der Nutzung Ihres Smartphones (d.h. für Telefonate, Nutzung von Apps)?

- ☐ Weniger als 30 Minuten.
- ☐ 30 bis 60 Minuten.
- ☐ 1 bis 2 Stunden
- ☐ Mehr als 2 Stunden.

Bitte schätzen Sie: Wieviele Apps nutzen Sie regelmäßig auf Ihrem Smartphone?

- ☐ Ich nutze gar keine Apps.
- ☐ Ich nutze nicht mehr als 5 Apps regelmäßig.
- ☐ Ich nutze nicht mehr als 10 Apps regelmäßig.
- ☐ Ich nutze mehr als 15 Apps regelmäßig.

Verwenden Sie Wearables? (z.B. Fitnessarmbänder wie Fitbit, Jawbone, Garmin etc.)

☐	☐
Ja	Nein

Bei Antwort Ja

Sie haben berichtet, dass Sie Wearables (z.B. Fitnessarmbänder) tragen. Zu welchen Anlässen tragen Sie diese Geräte?

- ☐ Nur zum Sport
- ☐ Nur tagsüber
- ☐ Nur nachts
- ☐ Sowohl am Tag als auch in der Nacht

Über welchen Zeitraum tragen Sie schon Wearables?

- ☐ erst einige Tage
- ☐ bereits einige Wochen
- ☐ schon einige Monate

Bei Antwort Nein

Könnten Sie sich vorstellen Wearables zu tragen und wenn ja wann? (z.B. Fitnessarmbänder wie Fitbit, Jawbone, Garmin etc.)

- ☐ Gar nicht
- ☐ Nur zum Sport
- ☐ Nur tagsüber
- ☐ Nur nachts
- ☐ Rund um die Uhr

5. **Bitte geben Sie an, welche Arten von Apps Sie auf Ihrem Smartphone verwenden. (Sie können mehrere Optionen auswählen.)**

- ☐ Apps zur Kommunikation (z.B. Whatsapp, Skype)
- ☐ Apps für Soziale Netzwerke (z.B. Facebook, Instagram)
- ☐ Fitness-, Gesundheits- oder Wellness-Apps (z.B. Runtastic, Freeletics)
- ☐ Unterhaltungs-Apps (z.B. Youtube, Spotify)
- ☐ Spiele-Apps (z.B. Quizduell, Temple Run)

Haben Sie bereits Smartphone-Apps genutzt, die sich speziell auf das Thema Depression bezogen haben?

☐ Nein, solche „Depressions-Apps“ habe ich noch nie benutzt.

☐ Ja, ich hatte bereits eine/mehrere „Depressions-App“ installiert, nutze diese aber momentan nicht mehr.

☐ Ja, ich habe eine/mehrere „Depressions-App“ installiert und nutze sie auch aktuell noch.

Bei Antwort Ja
Wenn Sie bereits eine/mehrere Depressions-Apps genutzt haben, welche waren das?

Wie intensiv haben Sie diese App verwendet?
Wenn Sie bereits mehrere Depressions-Apps ausprobiert haben, beantworten Sie die Frage bezogen auf die App, die Sie am intensivsten genutzt haben.

☐ Ich habe die App installiert und kurz angesehen, aber dann nicht verwendet.

☐ Ich habe die App nur für eine kurze Zeit (weniger als1 Monat) und unregelmäßig verwendet.

☐ Ich habe die App nur für eine kurze Zeit (weniger als1 Monat) aber regelmäßig verwendet.

☐ Ich habe die App längere Zeit (mind. 1 Monat), aber nur unregelmäßig verwendet.

☐ Ich habe die App längere Zeit regelmäßig verwendet.

☐ Keine dieser Beschreibungen trifft auf mich zu.

Welche Auswirkungen hat die Benutzung bei Ihnen gehabt?

☐ Ich habe positive Auswirkungen bemerkt.

☐ Ich habe sowohl positive als auch negative Auswirkungen bemerkt.

☐ Ich habe nur negative Auswirkungen bemerkt.

☐ Ich habe gar keine Auswirkungen bei mir bemerkt.

☐ Weiß nicht.

Wenn Sie positive Auswirkungen bemerkt haben, welche waren das?
Wenn die Frage nicht auf Sie zutrifft, oder Sie sich nicht erinnern können, lassen Sie das Feld einfach frei.

Wenn Sie negative Auswirkungen bemerkt haben, welche waren das?

Wenn die Frage nicht auf Sie zutrifft, oder Sie sich nicht erinnern können, lassen Sie das Feld einfach frei.

Bei Antwort Nein
Können Sie sich vorstellen, in Zukunft eine Depressions-App zu verwenden?

- ☐ Ja.
- ☐ Nein.
- ☐ Weiß nicht.

Was glauben Sie, welche positiven Auswirkungen könnte die Nutzung einer solchen Depressions-App möglicherweise haben?
Wenn Sie keine Meinung haben, lassen Sie das Feld bitte einfach frei.

Was glauben Sie, welche negativen/ungünstigen Auswirkungen könnte die Nutzung einer solchen Depressions-App möglicherweise haben?
Wenn Sie keine Meinung haben, lassen Sie das Feld bitte einfach frei.

6. **Welche der folgenden Funktionen würden Sie in einer Depressions-App nutzen:**
   Sie können mehrere Antwortalternativen auswählen.

- ☐ Dokumentation der Stimmung (über regelmäßige Selbsteinschätzungen)
- ☐ Dokumentation persönlicher Ziele für einzelne Tage oder Wochen (z.B. über Wochenpläne, Tagebuch-Funktion)
- ☐ Erfassung der Medikationseinnahme (über Medikamententagebücher)
- ☐ Messung der körperlichen Aktivität (z.B. über Erfassung von Schrittzahlen)
- ☐ Erfassung des Bewegungsradius (über GPS-Koordinaten)
- ☐ Messung der sozialen Interaktionen (z.B. über Anzahl eingehender und ausgehender Anrufe und sms-Nachrichten oder die Häufigkeit & Dauer der Nutzung von Kommunikations-Apps, wie Whatsapp)
- ☐ Erfassung des Schlafverhaltens (über Schlaftagebücher oder Schlaf-Sensoren)
- ☐ Erfassung von Stress- und Anspannungszuständen (z.B. über Messung von Herzraten, Körpertemperatur)

Welche Funktionen würden Sie sich für eine Depressions-App wünschen?
Wenn Sie keine Meinung haben, lassen Sie das Feld bitte einfach frei.

Wie viel Zeit würden Sie für die Nutzung einer Depressions-App pro Tag investieren?
Bitte schieben Sie den Regler auf eine geschätzte Anzahl Minuten.

7. **Smartphone-Apps bieten häufig auch die Möglichkeit, Daten mit Dritten zu teilen. Dies erlaubt zum Beispiel, sich mit anderen Nutzern zu vergleichen oder – bei Gesundheits-Apps – die Ergebnisse mit dem Hausarzt oder Therapeuten zu besprechen. Mit wem würden Sie die Daten einer Depressions-App teilen?**
   Sie können mehrere Antwortalternativen auswählen.

- ☐ Hausarzt
- ☐ Psychiater
- ☐ Psychotherapeut
- ☐ Familienangehörige
- ☐ Freunde/Bekannte
- ☐ Anderen Nutzer der App
- ☐ Forschungsprojekte zum Thema Depression (nur anonymisierte Daten)
- ☐ Meine Krankenkasse
- ☐ Niemandem der Genannten

Stellen Sie sich vor, Ihre Eingaben in der App deuten darauf hin, dass es Ihnen sehr schlecht geht. Wären Sie in einem solchen Fall damit einverstanden, dass die App automatisch jemanden über Ihren Zustand informiert?

- ☐ Ja, meinen Arzt oder Therapeuten.
- ☐ Ja, eine selbst gewählte Kontaktperson.
- ☐ Ja, einen Mitarbeiter den App-Anbieters.
- ☐ Nein.
- ☐ Weiß nicht.
